# The Analysis of Plastic Forming in the Rolling Process of Difficult-to-Deform Ti + Ni Layered Composites

**DOI:** 10.3390/ma18091926

**Published:** 2025-04-24

**Authors:** Dariusz Rydz, Sebastian Mróz, Piotr Szota, Grzegorz Stradomski, Tomasz Garstka, Tomasz Cyryl Dyl

**Affiliations:** 1 Faculty of Production Engineering and Materials Technology, Czestochowa University of Technology, 19 Armii Krajowej Avenue, 42-201 Czestochowa, Poland; sebastian.mroz@pcz.pl (S.M.); piotr.szota@pcz.pl (P.S.); grzegorz.stradomski@pcz.pl (G.S.); tomasz.garstka@pcz.pl (T.G.); 2 Department of Marine Maintenance, Faculty of Marine Engineering, Gdynia Maritime University, Morska Street 81-87, 81-225 Gdynia, Poland; t.dyl@wm.umg.edu.pl

**Keywords:** composite layered, asymmetric rolling, microstructure, Barkhausen noise

## Abstract

The article presents the results of experimental studies on the symmetrical and asymmetrical rolling process of composite laminate sheets consisting of difficult-to-deform Ti and Ni materials. Composite sheets joined by explosive welding were used for the tests. The aim of the research was to determine the impact of plastic shaping conditions in the rolling process on the quality and selected functional properties of the materials constituting the layered composite. The rolling process was carried out cold on a duo laboratory rolling mill with a roll diameter of 300 mm. During the rolling process, the influence of the rolling process conditions on the distribution of metal pressure forces on the rolls was determined, as well as the shear strength and microstructural studies of the joint area of the layered composites. As part of the conducted considerations, residual stress tests were carried out using the Barkhausen noise method. The scientific aim of the presented work was to determine the optimal conditions for the plastic processing of multi-layer Ti-Ni sheets. The results presented in the work allowed for determining the most favorable conditions for the rolling process.

## 1. Introduction

Homogeneous materials often do not meet the requirements of customers, such as the ability to work with high and variable loads, high temperatures, aggressive environments, resistance to abrasion, etc. [1,2]. There are many techniques that allow for selective impact on surface layers. One of them is burnishing [3,4], which is an extremely good cost-effective solution [5]. It has its limitations, but in such cases, coating technologies, including hybrid ones, are very good solutions [6,7,8]. However, hybrid coating technologies, although they give very good results, are usually expensive. A cost-effective and very smooth control is the plating technique, which results in layered composites [9,10]. Metal plating technologies have been known to mankind since ancient times. Although layered composites, including metal ones, have been known for a long time, they have only recently gained such extensive industrial applications [11,12]. Until the beginning of the 21st century, there was an increase in demand and intensive development of technologies for the production of multilayer materials. Multilayered products combining the properties of joined materials are nowadays an area of interest [13,14]. They are used, among others, in the chemical, energy, metallurgical, machine, and aviation industries [15,16].

There are relatively many methods of joining metals, such as explosive welding, surfacing, diffusion welding, friction welding, etc. Despite the continuous development of new solutions, it can be stated that the explosive welding (EW) method is still one of the most advantageous [17,18,19]. The explosive welding method, despite the increasingly common application, has not yet been fully understood [20,21,22]. The specific conditions prevailing during the joining process using the explosive welding method are characterized by very high pressure reaching thousands of atmospheres, extremely short process time (lasting up to several microseconds), and change in material density—they cause metals to join in physical states that do not correspond to the classical laws of mechanics [22,23]. However, the introduction of a new pair of materials into the rolling process due to the specificity of the process often causes many problems [24,25]. One of them is the occurrence of an intermetallic layer in the connection area [26]. This layer is particularly important during further plastic shaping due to its properties. These are usually hard and brittle materials that are exposed to micro cracks and fractures during deformation [27,28]. First of all, this is related to the uneven distribution of the individual layers deformation of composite sandwich sheets. This phenomenon results from differences in the mechanical properties of materials included in composite sheets, which was the subject of many works, including the authors of this publication [29,30].

Ti-Ni composite sandwich plates are used, among others, in the construction of electrolyzers for chlorine production. The electrolyzers made of Ti-Ni bimetal sheets are a battery of modules in which chlorine and hydrogen brine electrolysis takes place. Another example of the use of composite sandwich plates based on Ti-Ni sandwich plates is cladding plates used for heat exchangers. During the process of joining by the explosive welding method, during the detonation of explosives, there is a strong effect of both thermal and mechanical factors [2]. As a result of this interaction, structural changes occur in the joined materials and the area of direct connection. Another effect is the appearance of residual stresses in the joined materials, which very often results in deformation of the joined layered composite.

There are different techniques for joining Ti to Ni substrates, including brazing and diffusion bonding [31,32,33,34]. However, the diffusion bonding process requires a long holding time, usually more than 60 min, depending on materials, and a high temperature, which makes this method uneconomical [19,35]. Another method of joining metals and their alloys is the friction method. This method was one of the first to use friction as a solid-state welding technique [36].

The problem of uneven flow of layers can be solved by using an asymmetric rolling process [25,37,38]. This asymmetry can be imposed by differentiating the diameters of the working rolls or by differentiating the peripheral speeds of the working rolls. Asymmetry causes the activation of shear band broadening, which is found in mechanical properties [24,38]. Based on literature data [39,40] as well as the authors own publications [25,37], peripheral velocity asymmetry is used for both to control the curvature of the composite sheets and to reduce the metal pressure on the roll [2,25]. The first application is the correction of the bending defect of composite sandwich sheets after leaving the deformation gap [25,41]. In the second solution, reducing the metal pressure forces on the roller allows for the application of greater deformations in rolled sheets [24,42]. The rolling process carried out in this work is intended to determine the most favorable conditions for plastic forming of multilayer Ti-Ni sheets [39,40]. This work presents not only the results of research on deformations but also stresses occurring in the rolled multilayer sheets. The Barkhausen method [43] was used to analyze them. The results presented in the work will be of a utilitarian nature and will allow for their industrial application.

The aim of the work was to indicate the optimal conditions for the rolling process in terms of the quality of the joint area, microstructure, and curvature of multilayer sheets. The work presents the influence of the asymmetry of the working roll speed on both the curvature of multilayer sheets and the quality of the joint area of Ti-Ni layers. The research showed the presence of an intermediate layer in the joint area consisting of the aforementioned intermetallics. The rolling process was carried out on a rolling machine with the possibility of controlling the peripheral speed of the working rolls. The structural studies were conducted using optical microscopy and scanning microscopy. Non-destructive tests were also carried out using the Barkhausen noise (BN) method.

## 2. Materials and Methods

Tests of the symmetrical and asymmetrical rolling process were carried out on samples cut out from Ti-Ni sheets obtained using the explosive welding method. The process of explosive welding was carried out in the company ZTW EXPLOMET sp.j. Gałka, Szulc (Opole, Poland). Information about the parameters of the explosive welding process is confidential know-how of the company. The chemical composition of the tested materials is shown in Table 1.

**Figure 1:**
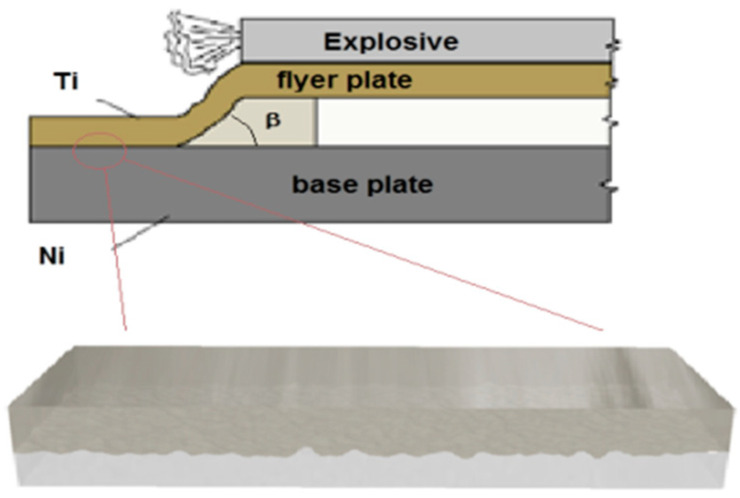
Scheme of plate joining by explosive welding of composite sandwich sheets Ti-Ni.

In order to be able to join sheets by explosive welding (Figure 1), the impact parameters must be met within which the welding process can be carried out. This range is most often presented in the coordinate system of the joining angle β and the joining speed.

Due to the fact that the process of manufacturing composite materials in this study, the following tests were performed:

- shear strength of the joint for materials before the rolling process;
- cold rolling;
- the residual stress analysis with the use of the non-destructive method using the Barkhausen noise (BN) technique,
- analysis of microstructure.

**Figure 2:**
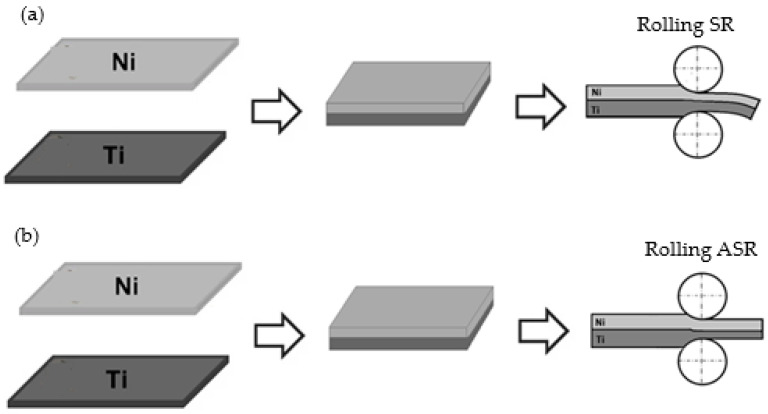
Diagram of the rolling process: (**a**) with equal peripheral speeds (SR process, V_U_ = V_L_), (**b**) asymmetry of peripheral speeds (ASR process, V_U_ < V_L_).

The rolling process of multilayer flat products is always a challenge. Due to the uneven deformation of the layers, uneven flow of the sheet metal layers is also observed in the plane of the exit from the rolling gap [2,36]. This phenomenon can be observed in Figure 2, where the rolling process is also presented.

As a part of this work, the process of rolling composite sandwich sheets from hard-deformable Ti-Ni materials was carried out. Rolling samples were cut out from the composite sandwich sheets obtained using explosion welding. The joined sheets had dimensions of 500 mm × 1200 mm, and the thickness of individual layers was Ti (grade 1) HTi = 1.0 mm and Ni (grade N0221) layers HNi = 1.0 mm. For the rolling process, specimens of 40 mm × 140 mm were used. Samples were cut out in the direction of propagation of the detonation wave. Tensile and shear tests were carried out using the Zwick Z100 testing machine (Ulm, Germany). Later on, the composite sandwich sheets rolling process was carried out. Rolling was performed for equal peripheral speeds of work rolls (symmetrical process—SR) and for kinetic asymmetry (asymmetrical process—ASR), where the peripheral speed of the roller in contact with the layer with higher deformation resistance was increased (in this case, the Ni layer). However, the controlled increase in the speed of the rolls in contact with the layer with greater resistance to plastic flow ensures a more even flow of metal, which makes it possible to obtain a straight composite sandwich sheet (Figure 2). The measurable value of this parameter is the values of the speed asymmetry factor of the peripheral working rolls, which can be written as [2]:

(1) $$a_{v}=\frac{V_{U}}{V_{L}}$$

where:

*V_U_*—peripheral speed of the upper roll,
*V_L_*—peripheral speed of the lower roll.

**Figure 3:**
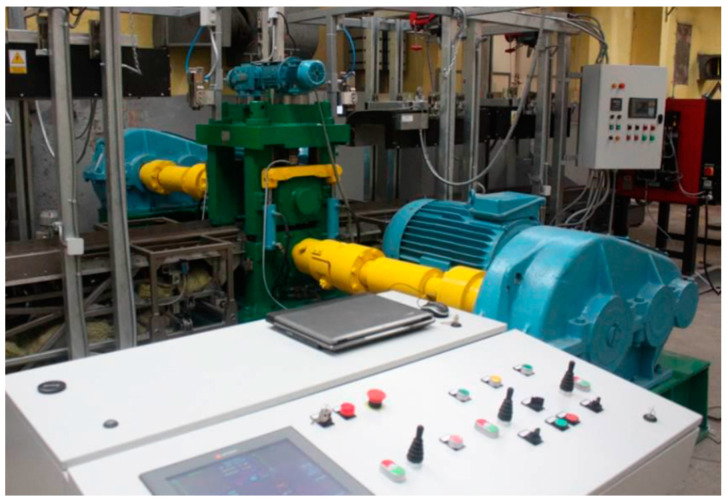
View of the 300 mm two-high laboratory mill used in the tests.

The rolling process was carried out on a semi-industrial two-high rolling mill with a roll diameter of 300 mm and individual drive for each roll (Figure 3) at an ambient temperature.

The rolling process was carried out for both equal (a_v_ = 1.0) and different (asymmetry) peripheral speeds of work rolls (a_v_ = 1.1). The value of the a_v_ parameter was taken from earlier work realized by authors [5,11]. The rolling speed was 0.2 m/s. The height of the rolling gap was set taking into account the stiffness of the stand. For the first two samples it was 1.5 mm, while for the next two it was 1.3 mm. Due to the fact that the rolling process was carried out at ambient temperature, which causes high metal pressure on the rolls, relative deformations were adopted for individual rolling variants: variant I ε = 10%, a_v_ = 1.0 and a_v_ = 1.1, variant II ε = 15%, a_v_ = 1.0, and a_v_ = 1.1. During the rolling process, measurements of force parameters were carried out. The presented test results are the results for one pass and show the difference in the values of layer deformation during the SR and ASR rolling processes.

Due to the fact that the rolling process was realized at ambient temperature, the authors decided to also analyze the residual stress state. According to earlier work of authors [24,42], the analysis was made with the use of the nondestructive technique, which is the measurement of Barkhausen noise. In the literature, the Barkhausen method is increasingly used to analyze the stress state resulting from various technological processes [44,45,46].

**Figure 4:**
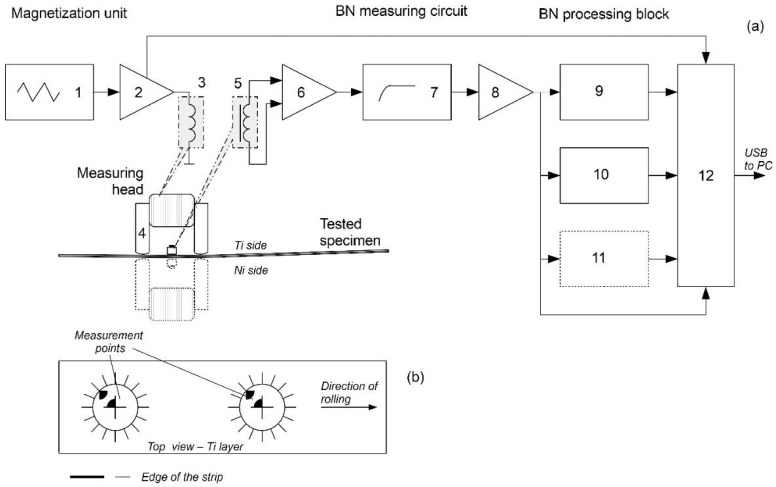
Schematic view of the experimental set-up: (**a**) functional block diagram, (**b**) a view of the measurement points, 1—RC generator, 2—current power amplifier, 3—magnetization windings, 4—yoke, 5—detection coil, 6—low-noise measuring preamplifier, 7—HP filter, 8—amplifier, 9, 10 and 11—processing block, 12—data acquisition card.

For measuring the Barkhausen noise’s parameters, special equipment for excitation, detection, and processing of BN has been developed [47]. Its functional block diagram is presented in Figure 4. The magnetization unit is based on a tuned triangle RC generator (1) and a current power amplifier (2). It supplies current Im (max. ±1.5 A) to the magnetization windings (3) wound on a yoke (4) of the measuring head. The BN signal excited in the tested material as an effect of cyclical magnetization is picked up by a detection coil (5) placed between poles of the yoke and next transmitted to the measuring block. The raw signal is amplified with the help of a specialized low-noise measuring preamplifier (6) with controlled gain ku1max up to 80 dB. Then, the signal is passed through a 1 kHz HP filter (7), where power line disturbances and harmonics of the magnetization current are eliminated. Finally, BN extracted from the background noise is gained by the amplifier (8) with ku2max = 40 dB and fed to a processing block. In the processing block, specialized circuits determine BN parameters: root-mean-square value (9), its envelope voltage signal (10), and, moreover, a series of the digital pulses corresponding to the Barkhausen jumps with amplitude over specific reference voltage (11), although this parameter was not analyzed in this study.

These signals, raw BN and the magnetization current in the form of a voltage signal, are acquired by a 1 MHz data acquisition card (12) type and sent via USB interface to the computer for further processing, saving, and displaying.

During the experiments in this study, the magnetization frequency fm = 12 Hz and 105 dB total gain of the measuring circuit were set. For the tests, a small measuring head was used, constructed on the base of a pocket of C-shaped segments made from transformer sheet and linear dimensions of 18 mm × 5 mm. The magnetization windings had 200 turns. The poles of the yoke were rounded faintly to obtain repeatable contact conditions with different curvature radii of the tested strip before as well as after rolling. Between the electromagnet poles, the detection coil wound on a small rectangular ferrite rod core with a cross-section of 6 mm^2^ was fixed elastically in a way that enabled slight vertical movement.

**Figure 5:**
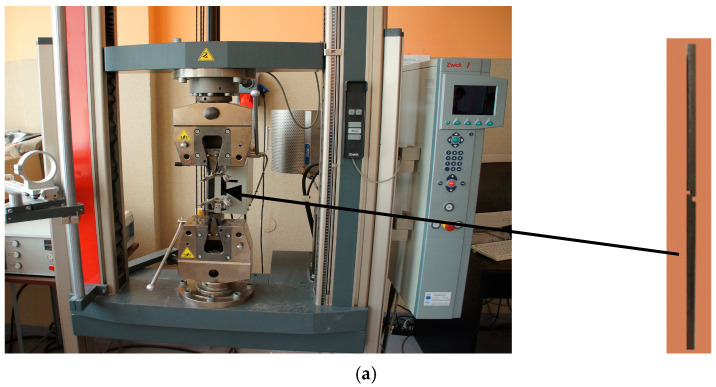

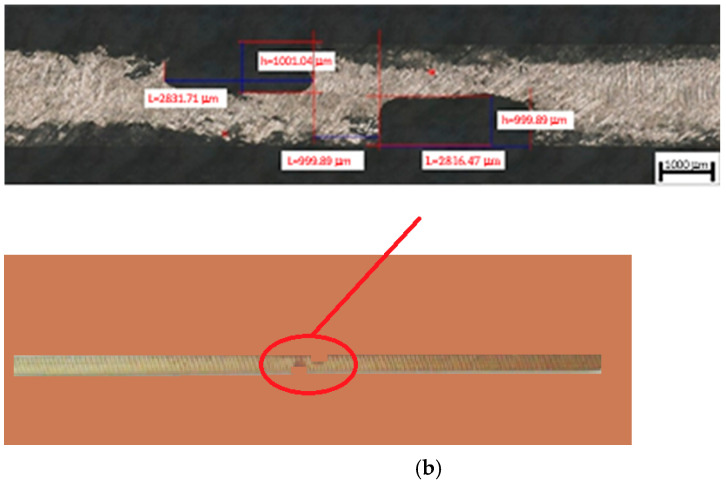
View of (**a**) the Zwick Z100 testing machine, (**b**) samples with measurement results.

On the surfaces of both sides of four Ti-Ni specimens (l = ~145 mm, w = 40 mm), two measurement points were marked by a permanent ink (Figure 5b). The distance between them was 60 mm, and the first point was signed 50 mm from the front of the specimens in the sense of the rolling start. In each measurement place, the lines of the measuring head positioning were drawn with angular step θ = 22.5°.

Due to the different orientation of the specimens during rolling, one of the edges of the strip was marked by the thick line too.

At these measurement points, the same set of directional BN measurements were done before and after the experiment of rolling Ti-Ni sheets. The measuring head position was changed between 0 and 180° with a constant step. The direction of the rolling pass line was assumed as the reference (0°).

In the final stage of research, the microstructure analysis was conducted. The analysis was conducted mainly for joining the area for material after explosive welding as well as after the rolling process. The research was conducted using the Nikon Eclipse Ma 200 producer microscope (Tokio, Japan) and Phenom XL (Thermo Fisher, Waltham, MA, USA) producer scanning microscope. During tests was also conducted the EDS analysis of the welding zone.

## 3. Results and Discussion

As part of the considerations, shearing tests of the joint areas were performed. Figure 5 shows a view of the sample before the process. In order to determine the cross-sectional area of the sheared samples, the length and width of the samples prepared for testing were measured.

The first stage of the conducted research on the bimetal after explosive welding considered mechanical properties of both layers as well as the joining area. Table 2 shows the properties of materials joined by explosive welding. The Ni layer was the base layer on which the Ti layer was shot.

In the process of joining materials with different properties by explosive welding (Table 2), a so-called intermediate layer is created, which is a mixture of the joined materials. Due to its different properties, unlike the joined materials, it often undergoes microcracks in the rolling process, which lead to a weakening of the strength of the joint area. Therefore, shear strength tests of the joint area are performed after each stage of the process of manufacturing flat-layered composites. Table 3 presents the results of the shear strength test of the Ni-Ti composite joint area. The results presented refer to the composites after direct explosive welding and after the symmetric and asymmetric rolling process.

The shear strength tests of the joint areas were carried out for the Ti-Ni material after direct connection (1), the ASR rolling process (2) for a relative reduction of ε = 10%, and after the SR rolling process (3) for a relative reduction of ε = 10%. The measurement results are presented in Table 3. Based on the conducted tests, it was observed that the rolling process affects the reduction in the shear strength properties of the connected Ti-Ni-layered composites. Based on the comparative analysis, it is clearly visible that the composite connection area is much less weakened in the case of using the ASR rolling process, which ensured obtaining straight Ti-Ni composite sheets after the deformation process. It should be emphasized that the value of the curvature of composite sheets is influenced by a number of both technological and material parameters. It depends to a large extent on the diversity of mechanical properties of the deformed materials [25]. Bending of composite sandwich sheets in the rolling process depends on many parameters, both technological and material properties. To a large extent, it depends on the diversity of mechanical properties of deformed materials [30].

**Figure 6:**
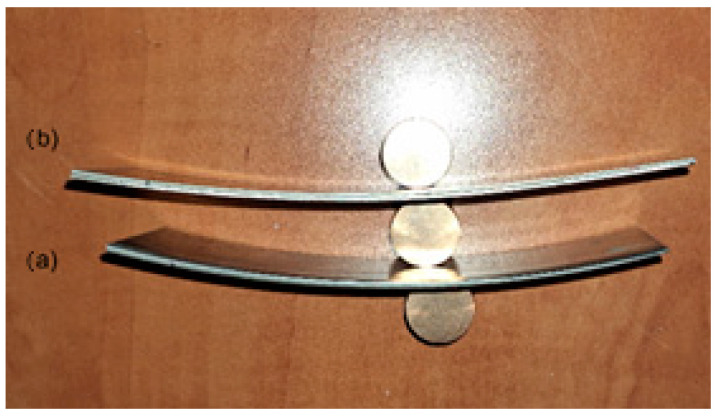
Shape of sample of Ti-Ni sheets after rolling: (**a**) ε = 10% a_v_ = 1.0 (No 1), (**b**) ε = 15%, a_v_ = 1.0 (No 3).

**Figure 7:**
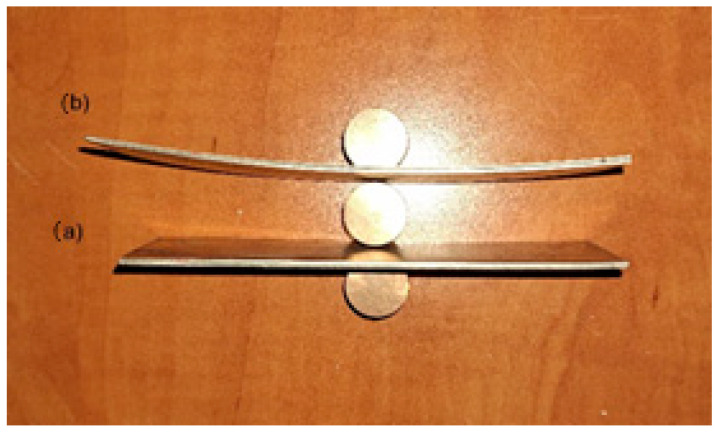
Shape of sample of Ti-Ni sheets after rolling: (**a**) ε = 15% a_v_ = 1.1 (No 4), (**b**) ε = 10%, a_v_ = 1.1 (No 2).

Figure 6 and Figure 7 show views of samples after symmetrical rolling (SR) and asymmetrical rolling (ASR) according to the scheme in Table 4.

**Figure 8:**
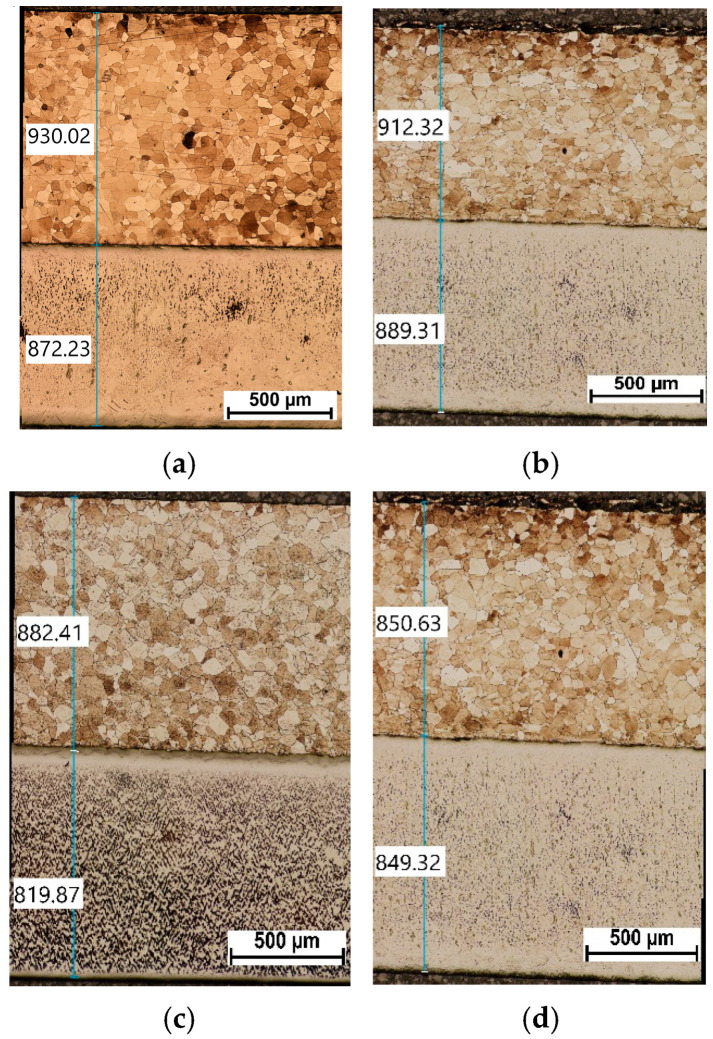
Optical microscopy view with connection areas and layer thickness measurements, (**a**) SR No. 1, (**b**) ASR No. 2, (**c**) SR No. 3, (**d**) ASR No. 4.

Figure 8 shows a view of the microstructure along with the thickness measurements of the individual layers (the result presented is the arithmetic mean of 5 measurements).

The results of rolling tests for individual variants are given in Table 4 and shown in Figure 6, Figure 7 and Figure 8. It should be emphasized that the value of the curvature of composite sheets is influenced by a number of both technological and material parameters. It depends to a large extent on the diversity of mechanical properties of the deformed materials [30]. Therefore, the use of new material pairs requires precise determination of the optimal forming conditions in the rolling process. In the considerations conducted for the ASR rolling variant, for the relative deformation ε = 10%, a straight strip was obtained. On the other hand, in the case of conducting the rolling process for the relative deformation ε = 15%, the introduced asymmetry had a positive effect on the shape of the rolled composite strip. However, the composite sheet still had a small bend, which is shown in Figure 7. The measured values of the strip curvature are given in Table 4, which clearly shows the beneficial effect of the ASR process on the curvature of the rolled Ti-Ni composite sheets.

**Figure 9:**
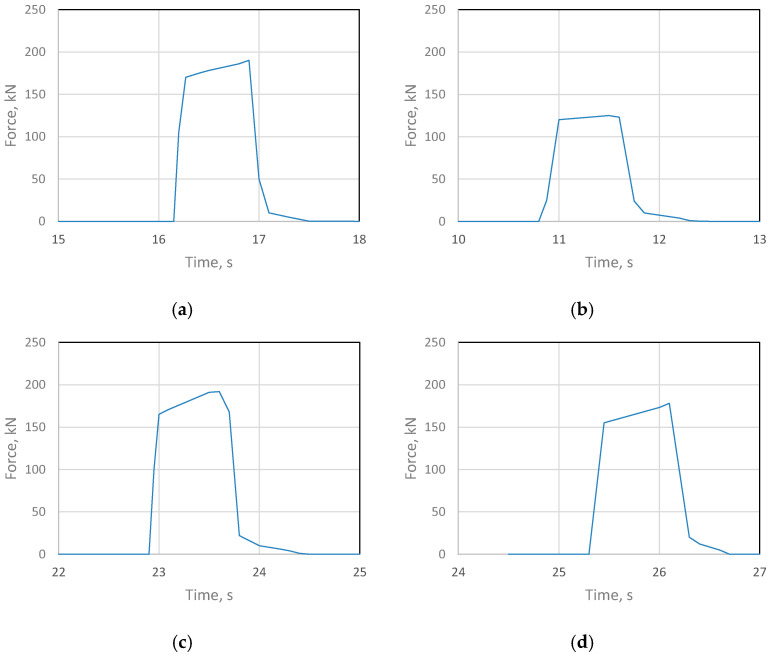
Changes in force on the rolls: (**a**) ε = 10%, a_v_ = 1.0; (**b**) ε = 1.0%, a_v_ = 1.1, (**c**) ε = 15%, a_v_ = 1.0, (**d**) ε = 15%, a_v_ = 1.1.

During the rolling process of composite sandwich sheets, the course of changes in metal pressure forces on the roll was measured and shown in Figure 9 as the sum of signals from both transducers.

The rolling process of composite sheets made of difficult-to-deform Ti-Ni materials differs from the research conducted so far in the field of manufacturing two-layer flat products. Analyzing the changes in the metal pressure force on the rolls, it can be seen that after introducing the asymmetry of the peripheral speed of the rolls, their value decreased. The reduction in the value of the metal pressure forces on the rolls as a result of using ASR is consistent with the literature assumptions [24]. The peripheral asymmetry of the work rolls introduced into the process was aimed at improving the shape of the composite sheets after the rolling process, which is shown in Figure 7 and Figure 8. The introduced difference in the peripheral speeds of the work rolls influenced the increase in the uniformity of deformation of the materials constituting the layered composite. Thus, the use of the asymmetry of the peripheral speeds also had a clear effect on the state of stress in the Ti-Ni composite materials. The results of stress tests in Ti-Ni-layered composites obtained by the Barkhausen method are presented in the further part of the paper.

**Figure 10:**
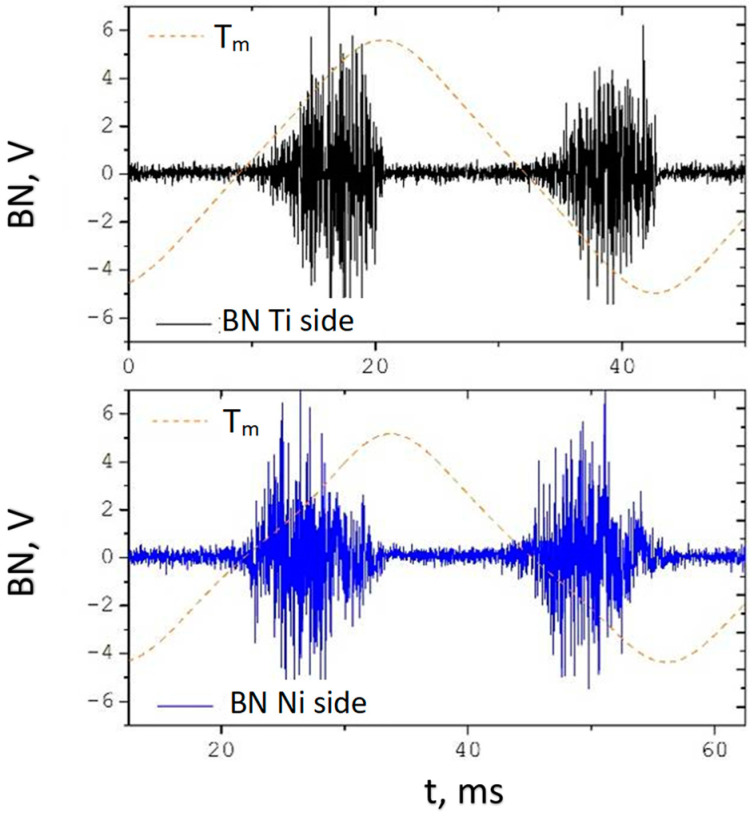
Barkhausen noise oscillograms picked up respectively at Ti and Ni sides for basic materials after explosive welding.

In the next stage of research, the residual stress analysis uses the Barkhausen noise (BN). Below are presented obtained results. Figure 10 shows the example oscillogram of BN captured at the Ni side and at the Ti side of composite sandwich sheets. BN measured through the Ti layer has a peak position (maximum of intensity) that is more delayed, indicating that at the side of the joint, the material is magnetically harder than on the other side (outer surface of the Ni layer). This bigger coercive force seems to be the result mainly of strong deformations of the nickel [24,44] caused by explosion and the presence of TiNi phases.

**Figure 11:**
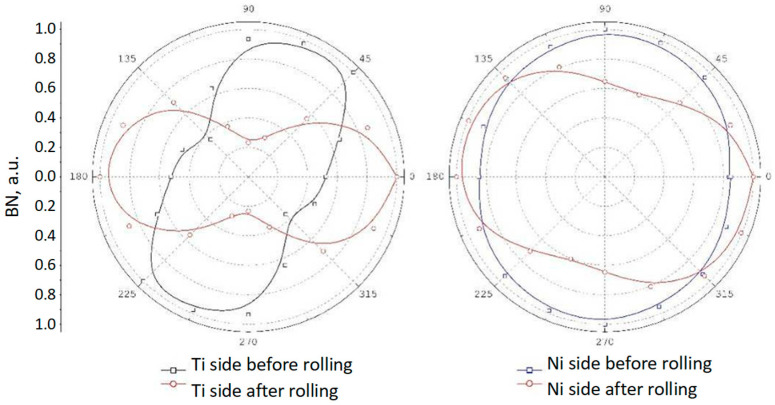
Examples of directional diagrams of BN intensity in measurement point no. 1.

Received results of investigations of BN intensity expressed by its RMS value and corresponding to the strain/stress state were shown in popular form in the directional diagrams. Due to different measurement and magnetization conditions during testing of both sides of the nickel layer, the data were normalized and presented in arbitrary units. For clear visibility of the principle axis of the pole figures, they were also drawn as B-splines. In Figure 11, representative directional diagrams in measurement point no. 1 (sample 1) are presented.

Before the rolling process, in the nickel layer, inside the sheet (measurements at the Ti side, black line), significant anisotropy of BN was observed, indicating the presence of an orthogonal biaxial stress state [45,48,49]. This layer was deformed by process of cladding, and, the main directions of strains at direction angles of 67.5° and 135° in this region result mainly from the direction of explosive wave propagation. At the outer side of the nickel not affected directly by the explosion (measurement at the Ni side, blue line), the shape of the radial plot points to a slightly biaxial stress state at directions 0° and 90°.

As can be observed, a significant influence of the rolling process on the re-orientation of BN intensity directional diagrams is identified (Figure 11, red lines). In this case, on both sides of the Ni layer, the main intensity of Barkhausen noise is in a direction parallel to the direction of rolling. It is an effect of the complex and strong cold deformation, affecting the magnetic domain structure of the nickel component too.

**Figure 12:**
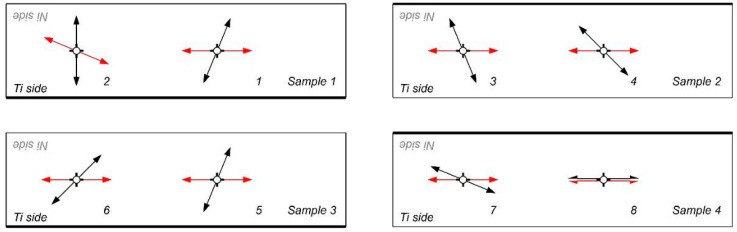
Orientation of the BN directional diagrams at Ti side of the sheet: before (black arrows) and after (red arrows) rolling process.

**Figure 13:**
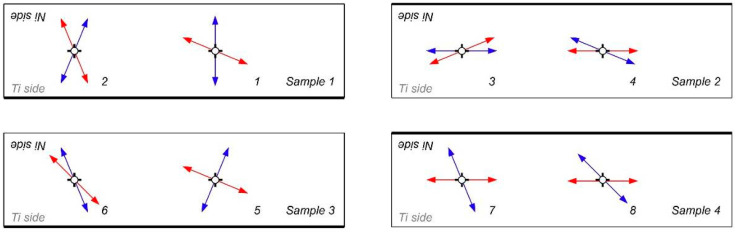
Orientation of the BN directional diagrams at Ni side of the sheet: before (blue arrows) and after (red arrows) rolling process.

In Figure 12 and Figure 13, extracted results of BN noise investigations in the other measurement points of tested samples were shown. For better understanding, on the sample shapes, the direction where the main intensity of the Barkhausen noise had occurred was marked by the arrows.

At first, samples after explosive welding were characterized by the totally different initial orientation of the direction of principle strains. Applying additional rolling processes caused that, in most cases, principle strains in samples were aligned to the direction near to the direction of rolling [49,50,51].

This effect is especially visible when rolling asymmetry has been applied. In this case, principle stress directions revealed by BN measurement on the Ni side (Figure 13) of the samples no. 2 and 4 were almost fully aligned with the direction of rolling. This proves the intensive deformation mechanism during this type of rolling.

Obtained results have confirmed the possibility of the stress and strain determination in the nickel layer of thin composite sandwich sheets by the Barkhausen method. It can be a new, additional, or supplementary testing tool for Ti-Ni sheets alongside existing ultrasound NDT of composite joint quality.

BN characterization can be useful not only for improving and developing the existing production technology but also for designing the manufacturing processes of the products from Ti-Ni sheets, considering the actual internal stress and strain state.

Due to the fact that deformation of EW materials in the merging area possesses stresses that in extreme cases can even lead to delamination, the authors have decided that microstructure analysis is necessary.

**Figure 14:**
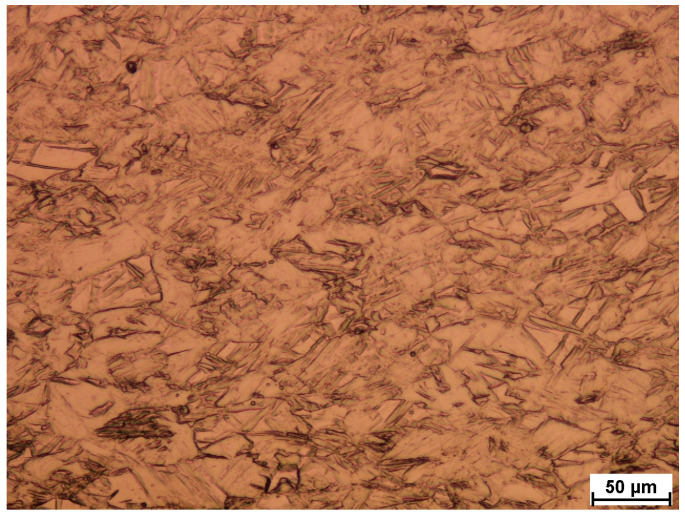
Ti layer (sheet) before welding, magn. 200×.

**Figure 15:**
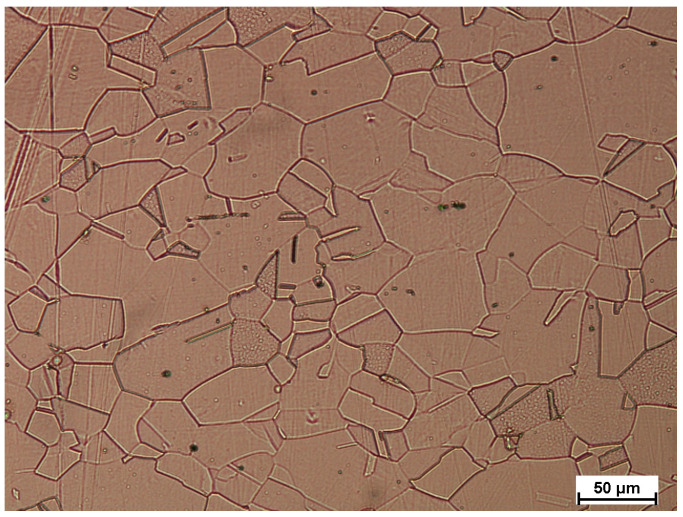
Ni layer (sheet) before welding, magn. 200×.

Therefore, in the last stage of research, the microstructure tests were conducted. Figure 14 presents the Ti layer (sheet) before welding; the material was etched (1 mL HF, 6 mL HNO_3_, 96 mL distilled water), and Figure 15 Ni layer (sheet) before welding (4 g copper sulfate, 20 mL HCl, 1 mL H_2_SO_4_, 16 mL distilled water). As it can be seen, there is no visible deformation of grain in both materials.

**Figure 16:**
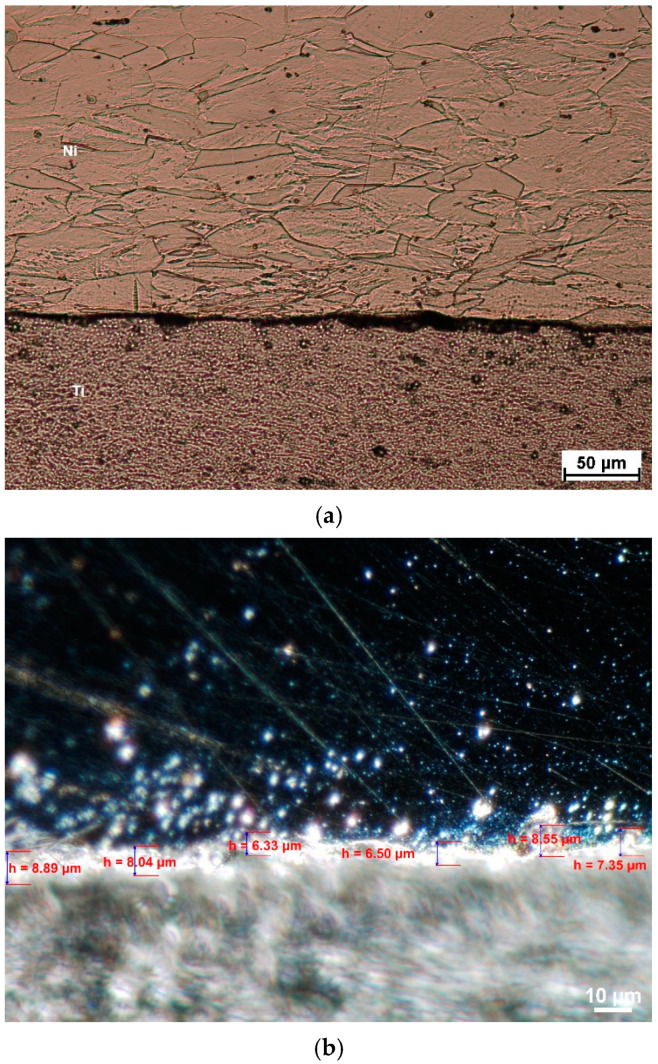
Microscopic view of the microstructure with image on the merging area with measurements of layers thickness, (**a**) raw state sample (after explosive welding) magn. 200×, (**b**) sample 1 (dark field) magn. 1000×.

The analysis on non-etched samples (Figure 16)—analysis of joining area quality and etched (4 g copper sulfate, 20 mL HCl, 1 mL H_2_SO_4_, 16 mL distilled water)—was conducted. The analysis of the possibility of the occurrence of discontinuities in the joining area on non-etched samples was made at 200 and 1000× magnification.

As can be seen, none of the samples show any traces of cracks in the area of the joint. This leads to the conclusion that the proposed welding technology is correct. However, attention should be paid to the presence of intermetallic phases and changes in the geometry of the merging area. The geometry changes are related to the rolling applied. As a result of plastic processing, the initial typical wavy connection was transformed into a desirable flat one.

After explosive welding, it can be seen that the Ni layer has an axial grain size from 80 to about 180 μm. Finer grains are located near the joint area. As a result of the applied plastic deformation, a negligible grain deformation can be observed along the rolling direction.

**Figure 17:**
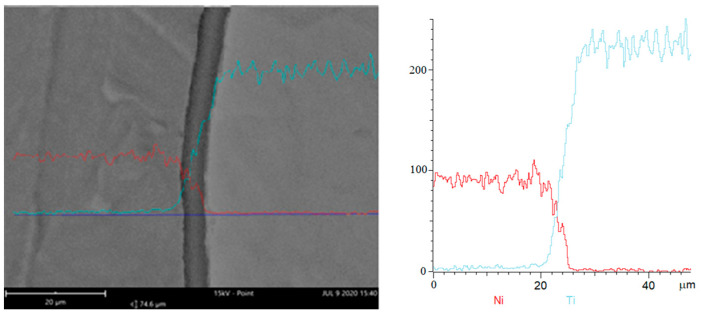
The samples EDS analysis of the joining area.

**Figure 18:**
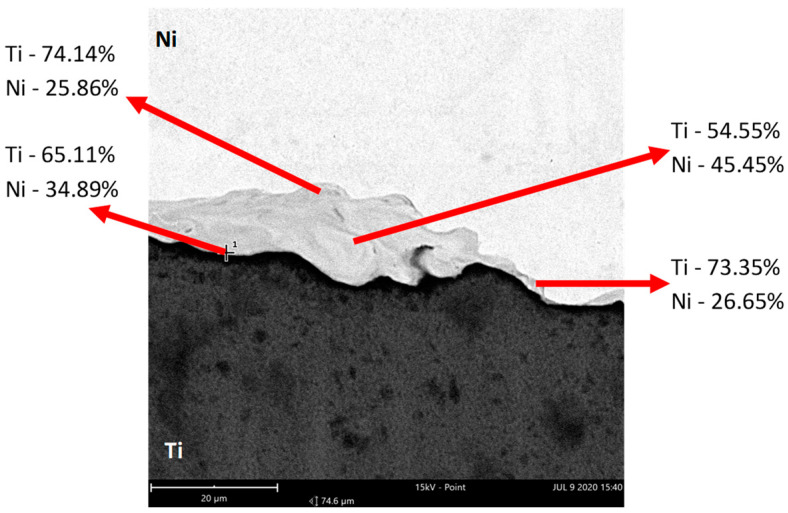
The point analysis in individual areas of the joining area.

**Figure 19:**
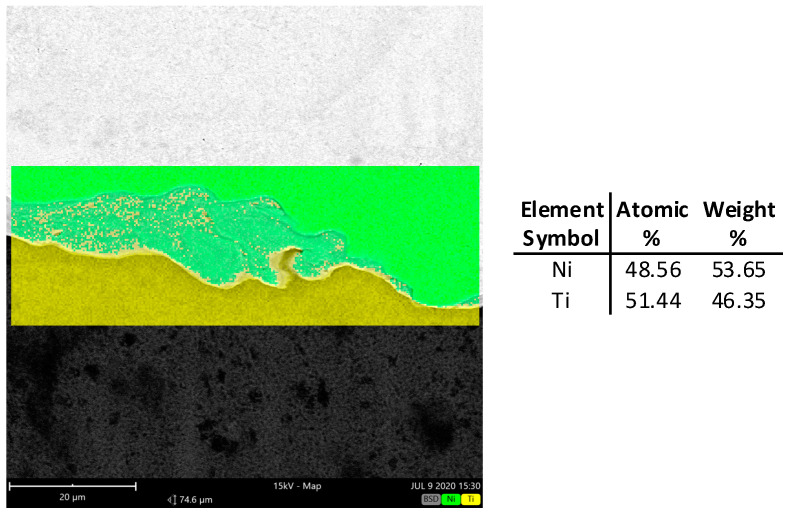
The BSD map of joining area with average chemical composition of the intermetallic layer.

Well visible is also the intermetallic, which analysis was made with use of the Phenom XL scanning microscope equipped with the EDS and BDS analyzer. Results of observation are presented in Figure 17 and Figure 18. The BSD analysis of the intermetallic layer, Figure 19, shows the map of elements observed in this area. Due to the fact that in the literature [21] it can be found that the joining area chemical composition is non-uniform and can be composed of the Ni_3_Ti, NiTi, and NiTi_2_ phases, point analysis in individual areas was performed.

As can be seen after microscopic analysis, the joining area is composed in part of an intermetallic layer. This area, however, is non-uniform (Figure 16); in the central part it has a composition of about 50% Ti and Ni, while in the zone close to the Ni layer it is 75/25% Ni/Ti. In the area near Ti, the proportion reverses, and it a composition of 65/35% and 73/27% Ti/Ni can be seen. What is interesting is that no crack in the joining area was observed for all analyzed samples. This confirms that the explosive welding technology and cold symmetrical and asymmetrical rolling are possible.

## 4. Conclusions

The cold-rolling process of sandwich composite sheets from hard-deformable Ti-Ni materials is complicated and difficult to develop. Based on the conducted experimental research, it can be concluded that the cold rolling process of composite sandwich sheets can be successfully used in industrial practice. The obtained results of experimental research allowed for the following conclusions to be formulated:

1. After the explosive welding process, a permanent connection was obtained with a shear strength more than three times higher than the requirements provided for by the standard—150 MPa.
2. During the rolling process, however, the problem is the selection of optimal process parameters for pairs of difficult-to-deform materials. The carried-out tests show that plastic deformation of Ti-Ni composite sandwich sheets were conducted properly. The introduction of asymmetry in the peripheral speed of the work rolls for rolling composite Ti-Ni sheets had a positive effect on their bending after the plastic forming process (Figure 6 and Figure 7). The macroscopic observation revealed that no cracks or delamination were observed in the area of the joint.
3. Obtained results have confirmed the possibility of the stress determination in the nickel layer of thin composite by the Barkhausen method. It can be a new, additional, or supplementary testing tool for Ti-Ni sheets alongside existing ultrasound NDT of composite joint quality.
4. The microstructure analysis of the joining area shows that cold deformation with the use of the symmetrical and asymmetrical rolling does not generate cracks. It was confirmed that the intermetallic layer is non-uniform. The chemical composition changes in volume. In the central part it is composed of a mixture of about 50% Ti and Ni.
5. It has to be stated that the intermetallic layer is not continuous; it can be seen that there are areas of intermetallics. The joining area after explosive welding is wavy; after the applied rolling process, the wave peaks slightly decrease. What is interesting is that the intermetallics after explosive welding are wider than after the rolling process.

## Figures and Tables

**Table 1:** Chemical composition of the used materials. weight %.

Material	Ti	Ni	Fe	N	C	Mn	Si	O
Ti (Grade 1)	balance	-	0.02	0.008	0.01	-	-	0.01
Ni (N0221)	-	balance	0.14	-	0.01	0.21	0.11	-

**Table 2:** Mechanical properties of materials used to combine into a composite.

Materials	UTS, Mpa	YS0.2, Mpa	HV_01_	A, %
Ti Grade 1	324	215	115	43
Ni N0221	390	170	95	48

Where: UTS—endurance limit, YS0.2—conventional endurance limit, HV_01_—hardness, A—lengthening.

**Table 3:** Shear strength results of the material after explosion welding and after the rolling process.

Sample	H mm	Dimensions of the Shear Area					Comments
		L mm	B mm	S mm^2^	Fmax kN	Rs Mpa	
1	2	1.02	20	20.4	9.2	450.8	rupture in the joining area
2	2	0.98	19.6	19.2	8.62	448.96	rupture in the joining area
3	1.8	0.95	20.2	19.19	8.37	436.16	rupture in the joining area for av = 1.0
4	1.8	1.07	20.1	21.51	9.64	446.92	rupture in the joining area for av = 1.1

Where: L, B, S—length, width, cross-sectional area of the sample for testing the shear strength of the joint.

**Table 4:** Results of the laboratory rolling tests SR and ASR.

No.	H_Ti0_/H_Ni0_ mm/mm	H_Ti_/H_Ni_ mm/mm	H_1_ mm	ε %	ε_Ti_ %	ε_Ni_ %	a_v_	1/R 1/m
1	1/1	0.87/0.93	1.8	10	13	7	1.0	1.18
**2**	**1/1**	**0.89/0.91**	**1.8**	**10**	**11**	**9**	**1.1**	**0.1**
3	1/1	0.82/0.88	1.7	15	18	12	1.0	0.98
4	1/1	0.85/0.85	1.7	15	15	15	1.1	0.44

## Data Availability

The original contributions presented in this study are included in the article. Further inquiries can be directed to the corresponding author.

## References

[1] Zhao Q., Hu X., Liu X. (2023). Analysis of Mechanical Parameters in Multi-Pass Asymmetrical Rolling of Strip by Slab Method. Materials.

[2] Rydz D. (2005). The Influence of Asymmetry Factor and Deformation on the Criterion Optimizing the Relative Flow Rate of St3S+0H13J Bimetallic Strip. Metalurgija.

[3] Grudzień J., Grochała D., Grzejda R., Kochmański P. (2024). Testing The Effectiveness of Hybrid Milling and Surface Burnishing in Improving the Wear Resistance of Machine Parts Made of Structural Steel. Lubricants.

[4] Grochała D., Grzejda R., Parus A., Berczyński S. (2024). The Wavelet Transform For Feature Extraction And Surface Roughness Evaluation After Micromachining. Coatings.

[5] Krajewski S.J., Grochała D., Tomków J., Grzejda R. (2023). Analysis of the Surface Stereometry of Alloyed Austenitic Steel After Fibre Laser Cutting Using Confocal Microscopy. Coatings.

[6] Strzelecki G.W., Nowakowska-Langier K., Namyślak K., Mulewska K., Wilczopolska M., Minikayev R., Nadolski M., Okrasa S., Romaniuk S., Zdunek K. (2025). Shannon Entropy Characterization of High-Entropy Thin Films Synthesized by Pulsed Magnetron Sputtering: The Influence of Modulation Frequency. Metall. Mater. Trans. A.

[7] Hawryluk M., Janik M., Zwierzchowski M., Lachowicz M.M., Krawczyk J. (2024). Possibilities of Increasing the Durability of Dies Used in the Extrusion Process of Valve Forgings from Chrome-Nickel Steel by Using Alternative Materials from Hot-Work Tool Steels. Materials.

[8] Krawczyk J., Łukaszek-Sołek A., Lisiecki Ł., Śleboda T., Hawryluk M. (2023). Wear Mechanisms of the Forging Tool Used in Pre-Forming in a Double Forging System of Truck Parts. Materials.

[9] Wang H., Chen N., Cheng H., Wang Y., Zhao D. (2024). Tensile Deformation Mechanism of an In Situ Formed Ti-Based Bulk Metallic Glass Composites. Materials.

[10] Eizadjou M., Kazemi Talachi A., Danesh Manesh H., Shakur Shahabi H., Janghorban K. (2008). Investigation of Structure and Mechanical Properties of Multi-Layered Al/Cu Composite Produced by Accumulative Roll Bonding (ARB) Process. Compos. Sci. Technol..

[11] Paul H., Faryna M., Prazmowski M., Banski R. (2011). Changes in the Bonding Zone of Explosively Welded Sheets. Arch. Metall. Mater..

[12] Zhao K., Xu D., Song X., Ma Y., Li H., Zhang J., Chen D. (2020). Reducing Yield Asymmetry between Tension and Compression by Fabricating ZK60/WE43 Bimetal Composites. Materials.

[13] Rydz D., Stradomski G., Pałęga M., Salwin M., Opydo M., Szarek A., Fik J., Chmielewski T. (2024). Analysis of Plastic Forming During Rolling of Al1050-AZ31-Al1050 Layered Composites for Transport Purposes. Adv. Sci. Technol. Res. J..

[14] Sang C., Cai X., Zhu L., Ren X., Niu G., Wang X., Feng P. (2020). Interfacial Microstructure of Ti/Ni Joints with Ti–Al Interlayer by Rapid Thermal Explosion Bonding in Vacuum. Vacuum.

[15] Nayanathara Thathsarani Pilapitiya P., Ratnayake A.S. (2024). The World of Plastic Waste: A review. Clean. Mater..

[16] Luo J., Aco V.L. (2004). Using cold roll bonding and annealing to process Ti/Al multi-layered composites from elemental foils. Mater. Sci. Eng. A.

[17] Mohanty A.K., Vivekanandhan S., Tripathi N., Roy P., Snowdon M.R., Drzal L.T., Misra M. (2023). Sustainable Composites for Lightweight and Flame Retardant Parts for Electric Vehicles to Boost Climate Benefits: A perspective. Compos. Part C Open Access.

[18] Fronczek D.M., Chulist R., Litynska-Dobrzynska L., Kac S., Schell N., Kania Z., Szulc Z., Wojewoda-Budka J. (2017). Microstructure and Kinetics of Intermetallic Phase Growth of Three-Layered A1050/AZ31/A1050 Clads Prepared by Explosive Welding Combined With Subsequent Annealing. Mater. Des..

[19] Tavoosi M. (2017). The Kirkendall Void Formation in Al/Ti Interface During Solid-State Reactive Diffusion Between Al and Ti. Surf. Interfaces.

[20] Wachowski M., Kosturek R., Śniezek L., Mróz S., Stefanik A., Szota P. (2020). The Effect of Post-Weld Hot-Rolling on the Properties of Explosively Welded Mg/Al/Ti Multilayer Composite. Materials.

[21] Topolski K., Wieciński P., Szulc Z., Gałka A., Garbacz H. (2014). Progress in the Characterization of Explosively Joined Ti/Ni Bimetals. Mater. Des..

[22] Karolczuk A., Kluger K., Derda S., Prazmowski M., Paul H. (2020). Influence of Impact Velocity on the Residual Stress, Tensile Strength, and Structural Properties of an Explosively Welded Composite Plate. Materials.

[23] Buque C., Tirschler W., Holste C. (1966). Analysis of Local Variations of Internal Stresses in Cyclically Deformed Nickel Crystal by Barkhausen Noise Measurements. Mat. Sc. Eng. A.

[24] Garstka T., Rydz D. (2015). Explosively Welded Ti-Ni Bimetallic Plate Characterization Using Barkhausen Noise. J. Electr. Eng..

[25] Rydz D., Dyja H., Berski S. (2003). The Prediction of Curvature of Bimetallic Plate Al-Cu During Asymmetrical Cold Rolling. Metalurgija.

[26] Mozaari A., Hosseini M., Manesh H.D. (2011). Al/Ni Metal Intermetallic Composite Produced by Accumulative Roll Bonding and Reaction Annealing. J. Alloys Compd..

[27] Gronostajski Z., Pater Z., Madej L., Gontarz A., Lisiecki L., Łukaszek-Sołek A., Łuksza J., Mróz S., Muskalski Z., Muzykiewicz W. (2019). Recent Development Trends in Metal Forming. Arch. Civ. Mech. Eng..

[28] Roberts I., Mynors D. (2012). Optimal Selection of Machine Parameters in Tension Leveling of Sheet Metals. Eng. Mater. Sci..

[29] Mihara-Narita M., Asai K., Sato H., Watanabe Y., Nakatsugawa I., Saito N., Chino Y. (2025). Dissimilar Welding of Magnesium Alloys and Aluminum Alloys by Explosive Welding. Materials.

[30] Wierzba A., Mróz S., Szota P., Stefanik A., Mola R. (2015). The Influence of the Asymmetric ARB Process on the Properties of Al-Mg-Al Multi-Layer Sheets. Arch. Metall. Mater..

[31] Sun H., Li Q., Chan Y.C. (2014). A Study Of Ag Additive Methods by Comparing Mechanical Properties Between Sn57.6Bi0.4Ag and 0.4wt% Nano-Ag-Doped Sn58Bi BGA Solder Joints. J. Mater. Sci. Mater. Electron..

[32] Cao J., Li C., Qi J., Shi Y., Feng J. (2015). Combustion joining of carbon–carbon composites to TiAl intermetallics using a Ti–Al–C powder composite interlayer. Compos. Sci. Technol..

[33] Zdrodowska K., Wojsyk K., Szala M. (2014). The Microstructural Properties of Explosion Welded Ni/Ti Joint. Adv. Sci. Technol. Res. J..

[34] Zhang B., Zhang Z., Zhao T., Zhang D., Cheng J., Wang W., Qiao K., Yang Y., Wang K. (2019). Effect of Temperature on the Interfacial Evolution of Ti/Ni Multilayered Composites Fabricated by ARB. J. Alloys Comp..

[35] Shen T.D., Quan M.X., Wang J.T. (1993). Solid State Amorphization Reactions in Ni/Ti Multilayer Composites Prepared by Cold Rolling. J. Mat. Sc..

[36] Gavalec M., Barenyi I., Krbata M., Kohutiar M., Balos S., Pecanac M. (2023). The Effect of Rotary Friction Welding Conditions on the Microstructure and Mechanical Properties of Ti6Al4V Titanium Alloy Welds. Materials.

[37] Grassino J., Vedani M., Vimercati G., Zanella G. (2017). Effects of Skin Pass Rolling Parameters on Mechanical Properties of Steels. Int. J. Precis. Eng. Mnanuf..

[38] Zhao J., Wang X., Yang Q., Wang Q., Wang Y., Li W. (2021). Mechanism of Lateral Metal Flow on Residual Stress Distribution During Hot Strip Rolling. J. Mater. Proces. Tech..

[39] Mróz S., Szota P., Stefanik A. (2016). The Theoretical and Experimental Analysis of the Possibility of Employing the Groove Rolling Process for the Manufacture of Mg/Al Bimetallic Bars. Metalurgija.

[40] Abvabi A. (2014). Effect of Residual Stresses in Roll Forming Process of Metal Sheets. Ph.D. Thesis.

[41] Skripalenko M.M., Galkin S.P., Karpov B.V., Romantsev B.A., Kaputkina L.M., Danilin A.V., Skripalenko M.N., Patrin P.V. (2019). Forming Features and Properties of Titanium Alloy Billets After Radial-Shear Rolling. Materials.

[42] Naizabekov A., Lezhnev S., Arbuz A., Panin E. (2018). The Effect of Radial-Shear Rolling on Microstructure and Mechanical Properties of Stainless Austenitic Steel AISI-321. MATEC Web Conf..

[43] Garstka T. The Complex System for Residual Stress Determination Based on Barkhausen Noise Measurement. Proceedings of the 5th International Conference on Barkhausen Noise and Micromagnetic Testing.

[44] Pitoňák M., Mičietová A., Moravec J., Čapek J., Neslušan M., Ganev N. (2024). Influence of Strain Rate on Barkhausen Noise in Trip Steel. Materials.

[45] Sheng H., Wang P., Yang Y., Tang C. (2024). Stress and Microstructures Characterization Based on Magnetic Incremental Permeability and Magnetic Barkhausen Noise Techniques. Materials.

[46] Wu H., Ziman J.A., Raghuraman S.R., Nebel J.-E., Weber F., Starke P. (2023). Short-Time Fatigue Life Estimation for Heat Treated Low Carbon Steels by Applying Electrical Resistance and Magnetic Barkhausen Noise. Materials.

[47] Nishihara H., Taniguchi S., Maeda H., Oguro I., Harada M., Ogino T., Matsumoto S., Shindo Y., Ohtsuka S. The effect of mechanical stress on Barkhausen noises from heat-treated nickel plates. Proceedings of the 12th Asia-Pacific Conference on NDT.

[48] Vengrinovich V., Tsukerman V.L. Stress and Texture Measurement Using Barkhausen Noise and Angular Scanning of Driving Magnetic Field. Proceedings of the 15th WCDNT—World Conference on Nondestructive Testing.

[49] Piotrowski L., Chmielewski M., Augustyniak M., Maciakowski P., Prokop K. (2015). Stress Anisotropy Characterisation with Help of Barkhausen Effect Detector with Adjustable Magnetic Field Direction. Int. J. Ap. Electr. Mech..

[50] Silva S., Mansur T., Palma E. Determining Residual Stress in Ferromagnetic Materials by Barkhausen Noise Measurement. Proceedings of the 15th World Conference on Nondestructive Testing.

[51] Augustyniak B. (2003). Magnetomechanical Effects and Their Applications at Non-Destructive Evaluation of Materials. Ph.D. Thesis.

